# Uncoupling Traditional Functionalities of Metastasis: The Parting of Ways with Real-Time Assays

**DOI:** 10.3390/jcm8070941

**Published:** 2019-06-28

**Authors:** Sagar S. Varankar, Sharmila A. Bapat

**Affiliations:** National Centre for Cell Science, Savitribai Phule Pune University, Ganeshkhind, Pune 411007, India

**Keywords:** metastasis, functional read-outs, metastatic modalities, live cell imaging, quantitative metrics

## Abstract

The experimental evaluation of metastasis overly focuses on the gain of migratory and invasive properties, while disregarding the contributions of cellular plasticity, extra-cellular matrix heterogeneity, niche interactions, and tissue architecture. Traditional cell-based assays often restrict the inclusion of these processes and warrant the implementation of approaches that provide an enhanced spatiotemporal resolution of the metastatic cascade. Time lapse imaging represents such an underutilized approach in cancer biology, especially in the context of disease progression. The inclusion of time lapse microscopy and microfluidic devices in routine assays has recently discerned several nuances of the metastatic cascade. Our review emphasizes that a complete comprehension of metastasis in view of evolving ideologies necessitates (i) the use of appropriate, context-specific assays and understanding their inherent limitations; (ii) cautious derivation of inferences to avoid erroneous/overestimated clinical extrapolations; (iii) corroboration between multiple assay outputs to gauge metastatic potential; and (iv) the development of protocols with improved in situ implications. We further believe that the adoption of improved quantitative approaches in these assays can generate predictive algorithms that may expedite therapeutic strategies targeting metastasis via the development of disease relevant model systems. Such approaches could potentiate the restructuring of the cancer metastasis paradigm through an emphasis on the development of next-generation real-time assays.

## 1. Introduction

Metastasis generates a systemic disease driven by the concerted alliance of tumor cell dissociation, physical translocation, and distant colonization. The dissociation of cells from the primary tumor initiates the metastatic cascade and generates distinct entities defined by severance dynamics of cell adhesion complexes. Further, acquisition of migratory/invasive properties by tumor cells facilitates their entry into circulation (or lymphatic system), wherein they evade host immune responses and endure extrinsic pressures followed by extravasation at secondary site(s). Tumor cell dormancy and secondary site remodeling then define the latency of colonization, which eventually establishes metastatic lesions [1,2,3]. An intricate interplay of molecular networks drives these programs and contributes to the efficacy of tumor progression [1,2,3,4]. Phenotypic switches induced by epithelial–mesenchymal transitions (EMT) are widely studied, and are deemed crucial for successful metastasis. Alternatively, recent studies highlight a collective mode of dissemination, wherein the retention of several epithelial properties is well documented [1,2,3,4]. These processes are further influenced by numerous physiological parameters, including the tissue stroma, architecture, and extra-cellular matrix (ECM) composition, in order to facilitate the successful establishment of a secondary disease [1,2,3,4]. Thus, collaboration amongst diverse functionalities outlines a complex blueprint of the process, wherein distinct modes of tumor cell dissemination can influence disease aggressiveness and therapeutic response.

Routine analyses in cancer metastasis studies inadvertently employ endpoint or “snapshot” assays that widely focus on the physical translocation of cancer cells. Functional read-outs tend to simplify various aspects of metastasis, and often neglect the role of tumor heterogeneity in disease progression. Furthermore, current notions extensively associate dissemination with a mesenchymal phenotype, and disregard the contributions of the alternate processes that are often unnoticed by existing experimental systems [3,5,6]. Detailed examination of the metastatic cascade thus necessitates the integration of the recent conceptual and technical advances for the development of informative assay protocols. In this article, we present a reassessment of the functional read-outs routinely employed in metastasis-associated studies, and accentuate the application of high-resolution imaging approaches to derive relevant patho-physiological conclusions.

## 2. Snapshot Assays for Metastasis Assessment

**Figure 1:**
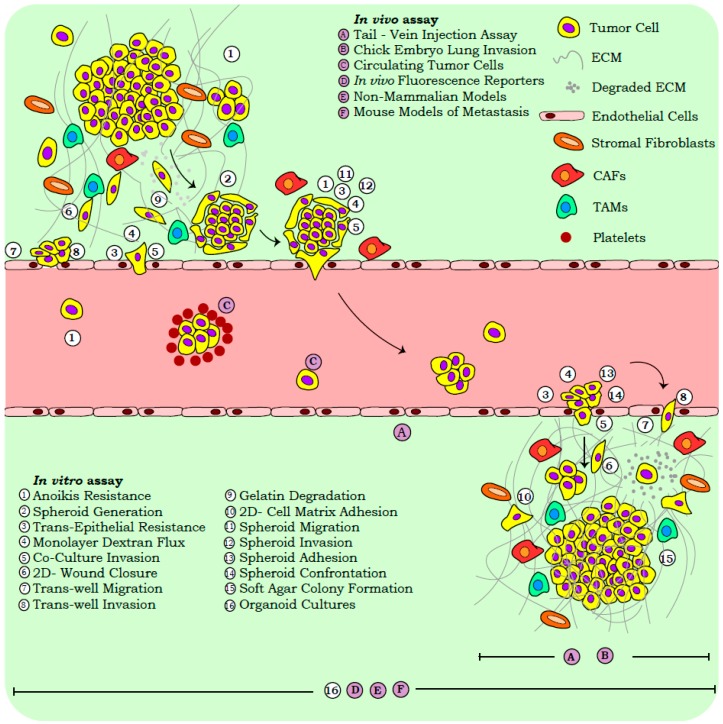
Functional assays for the metastatic cascade. Metastasis encompasses three distinct stages, *viz.* primary dissociation, physical translocation, and colonization. The interplay of complex processes severs cells from the primary tumor; these cells proliferate, migrate, and invade through the tissue matrix to initiate hematogenous or lymphatic dissemination. Circulating tumor cells then overcome hydrostatic pressures and immune surveillance to extravasate and colonize distant tissues to seed micro-/macro-metastases. Diverse cellular functions activated during the metastatic cascade are evaluated experimentally by functional assays, and can be modified to accommodate multiple biological components (micro-environmental milieu, extra-cellular matrix–ECM, stromal cells, extrinsic physical pressures, immune cells, and so on). A list of the relevant assays employed across the metastatic cascade are listed and indicated in the schematic.

Reliance on snapshot assays in cancer research is accounted for by several advantages, including the ease of execution, reproducibility, and applicability in high throughput screens. In vitro functional read-outs provide a preliminary assessment of the metastatic capabilities, while the variables contributing to tumor heterogeneity viz., micro-environmental milieu, tissue specific metabolic gradients, and systemic architecture, are often assessed with in vivo models. While our review focusses on the widely employed functional assays corresponding to three distinct stages of metastasis, *viz.*, primary dissociation, physical translocation, and colonization, we briefly discuss the molecular approaches routinely used in cancer biology (Figure 1).

### 2.1. Molecular Assays

The functional assessment of metastasis is often correlated with the molecular signatures derived from tumor cells or cell line models. Primary profiling studies employ a wide range of markers identified across the metastatic cascade, which include cell junction and cytoskeletal components, transcription factors (TFs), secretory enzymes, and cell surface receptors [7]. Molecular profiles, averaged from a cell population, can often misrepresent disease heterogeneity, as affirmed by the reports on single cell characterization, besides over-emphasizing the role of EMT during metastasis [8,9,10]. Microscopy studies further associate the sub-cellular localization of several phenotype associated markers and TFs with distinct cellular functions [11,12]. Importantly, recent reports associating altered marker sub-cellular localization with pathological conditions necessitate the inclusion of this parameter in clinical assessments [13,14,15]. Furthermore, mechanistic studies on cell state maintenance employ fluorescence or enzyme (luciferase, β-galactosidase, and chloramphenicol acetyltransferase) assisted reporter systems for quantifying gene regulation [16,17]. Apart from the static molecular profiles, cytoskeletal, vesicular, and membrane dynamics, as captured by microscopy, offer deeper insight into the alterations of the cell shape and function [18,19,20].

Molecular assays, however, rely on markers that often exhibit extensive disparities across model systems, and are subject to cellular context-specific modulation [21,22,23]. E-cadherin expression and membrane localization, often gauged in clinical specimens by immuno-histochemical scoring, were exclusively associated with the lack of metastasis [24,25,26]; however, the detection of this adherens junction molecule in collectively metastasizing cells challenges its inverse correlation with dissemination [27,28]. Recently, E-cadherin negative cells have also been reported to exhibit collective migration by virtue of CD44 mediated cell-cell adhesion in invasive breast lobular carcinoma [29]. Such discrepancies arise from tissue-specific plasticity programs that are influenced by the local microenvironment. Similarly, the divergent contribution of regulatory TFs in metastasis has been reported; some examples include the stage specific roles of the EMT-mediating TFs Zeb1 and Zeb2 in pancreatic cancer and melanoma dissemination [30,31]; an EMT-TF circuitry switch in melanoma, wherein Slug–Zeb2 act as tumor suppressors in melanocytes, while Twist1–Zeb1 function towards neoplastic transformation [32]; the tissue-specific expression of the Prrx1 isoforms (Prrx1a and Prrx1b) that govern the distinct phenotypic states in pancreatic and breast cancer progression [33,34]; the co-operative role of Slug and Sox9 in the maintenance of breast epithelium homeostasis [35]; and so on. Thus, assigning relevance to metastases signatures requires an accompanying physiological comprehension of the cellular plasticity, and corroboration with tissue specific molecular profiles, mechanistic approaches, and imaging protocols.

### 2.2. Functional Assays

The examination of functionalities across the physiological and pathological states is robustly aided by cell-based assays. Routinely employed assays in cancer biology gauge the properties of anoikis resistance, stemness, migration, invasion, and colonization, so as to correlate with clinical observations (Table S1 and Figure 1).

#### 2.2.1. In Vitro Assays

The loss of cell–cell and cell–matrix contacts initiates the anoikis cascade under physiological conditions, whereas resistance to this cell-death pathway in tumor cells permits effective disease progression [36]. The in vitro assessment of “anoikis” relies on the differential uptake of fluorescent dyes or the biochemical conversions of fluorophores by viable versus non-viable cells. Dissociated tumor cells exhibit a tendency to generate organized multi-cellular structures termed “spheroids”, disorganized “cellular aggregates”, or they exist as single cells [37]. Cell aggregates and spheroids exhibit stem-like and anoikis resistance properties, and are associated with disease aggressiveness [38,39].

The functional attributes of cell migration and invasion are also hijacked during tumor progression. Routine assessment of migratory capabilities by the “in vitro wound closure assays” is often enhanced by the fluorescent tagging of cells with reporter proteins or membrane labelling dyes [40,41]. The efficacy of tumor cell intra-/extra-vasation is recapitulated in vitro by trans-well inserts of pre-defined pore sizes. Such assays are employed widely to quantify “cell motility” in response to a chemotactic agent, while the layering of artificial matrix components (such as matrigel, carboxy methyl cellulose, and hydrogels) over these inserts gauge properties of “cell invasion” and “ECM remodeling” [38]. Recent trans-well systems employ micro-electrode coupled inserts that measure the impedance flux in response to cell migration/invasion, with enhanced precision [42]. Tumor cell invasion also entails the biochemical and mechanical modification of ECM components. “ECM degradation assays” measure the enzymatic activity of secreted matrix metalloproteinases (MMPs) by employing fluorescently labelled substrates and quantifying the signal intensity in the vicinity of invasive tumor cells [43]. Additional information pertaining to tumor cell invasion is gained from the “co-culture assays” that recapitulate the in-situ disruption of non-transformed tissue linings often encountered by metastasizing cancer cells [44]. The use of differentially labelled non-transformed and cancer cells improves the resolution of this assay, by enhancing the visualization and quantification of monolayer perturbance and invasion by tumor cells. Monolayer disruption by invading cancer cells is also quantified by the “trans-epithelial resistance (TER)” and “dextran flux assays” that gauge monolayer integrity and permeability, respectively [45,46]. Recent developments in advanced microfluidic and flow cytometry approaches have also facilitated the isolation of “circulating tumor cells (CTCs)”, which are actual proof of an ongoing metastatic cascade [10,47]. Several recent reviews detail, at length, the role of CTCs in tumor biology and translational medicine, and associate them with stem-like properties, immune evasion signatures, and immense phenotypic plasticity [48,49,50]. CTCs also offer excellent diagnostic/prognostic value and specific targeting opportunities [51,52,53]. Hence, their detection, quantification, and analyses are being developed in view of clinical applications.

Cell-substrate adhesion, governed by tissue specific ECM components, represents a critical determinant of metastatic seeding [54,55,56]. The in vitro assessment of “adhesion” employs ECM pre-coated plates to identify the critical molecular players mediating cell–matrix interactions [57]. Separately, anchorage independent growth of tumor cells as gauged by the “soft agar assay” utilizes a three-dimensional (3D) matrix devoid of ECM components, and quantifies the tumor seeding capacity in vitro [58]. Apart from studies pertaining to single cell colonization, spheroids and cell aggregates are also functionally examined for their properties of adhesion, migration, and invasion, which can be extrapolated to the metastatic cascade [38]. Furthermore, the competitive interaction of these entities in suspension is assessed by “spheroid confrontation”; such an assay evaluates the differential invasive capabilities, as well as co-operation between these cell populations in view of metastases seeding [38].

Despite their obvious advantages, cell line models provide limited information on the in situ landscape of a disease, because of a lack of higher order organization conferred by the tissue architecture. “Organoid cultures” represent an in vitro 3D model system reminiscent of the in-situ organization, and provide improved clinical correlations [59]. The sustenance of genetic features from patient samples by organoids make them improved models for studying metastases as opposed to cell lines, which can often accumulate genetic aberrations over multiple passages [60]. Their amenability to in vitro functional assays further permits a high throughput assessment of pathological states. Studies with pancreatic and colorectal cancer organoids, established from clinical specimens, demonstrate the recapitulation of histological features reminiscent of the parental tissue, when injected into immunocompromised mice [61,62]. Interestingly, stage-specific organoids have also captured the evolving heterogeneity and molecular landscapes of tumors, and can improve the efficacy of therapeutic interventions in personalized medicine [63]. Similarly, organoids have been developed from CTCs that exhibit drug-resistance responses similar to the patients, and are amenable to high-throughput screening for the design of personalized therapeutic regimes [64]. Additional details on the advancements in organoid generation and utility in cancer biology are stated elsewhere [64,65,66].

#### 2.2.2. In Vivo Systems

Despite the widespread applicability of in vitro systems, several aspects of metastasis that contribute to its complexity and heterogeneity are comprehended only when examined in vivo. Several non-mammalian systems have been extensively studied so as to comprehend the functionalities associated with the metastatic cascade. Examples include chemotactic migration observed in *Dictyostelium discoideum*, anchor cell invasion documented during vulva morphogenesis in *Caenorrhabditis elegans*, collective cell migration of ovarian border cells in *Drosophila melanogaster,* and the amoeboid migratory phenotype associated with primordial germ cells of *Danio rerio*. Extensive details on these non-mammalian models have been documented elsewhere [67]. Similarly, the developing chick embryo has been utilized for the assessment of its metastatic capabilities. The traditional chick “chorioallantoic membrane” assay quantifies tumor cell invasion across embryonic layers, and is assessed microscopically [68,69]. Recent “ex ovo embryo-xenograft models”, however, serve as improved visual and quantitative systems for metastatic dissemination and intra-vital imaging [69]. Despite the evolutionary divergence, the conservation of distinct cellular functionalities in these animal models, along with their amenability to genetic screens and live cell imaging, permit the extrapolation of relevant observations to mammalian systems. However, the routine and widespread utility of mouse models in cancer biology as opposed to non-mammalian systems continues, because of emphasis on the clinically relevant settings that are more effectively mimicked by genetically engineered mice.

“Mouse models” recapitulate physiological variables contributing to disease progression, which are absent in vitro and allow for the derivation of clinically relevant outputs. We describe briefly a few mouse models and assays employed in tumor biology; interested readers may refer to other detailed articles [70,71,72]. The metastatic cascade is routinely captured by spontaneous models for metastasis that involves ectopic/orthotopic tumor cell transplantation in immunocompromised mice. Orthotopic models more effectively represent disease progression, as they expose tumor cells to the micro-environmental cues encountered in the tissue of origin. Alternatively, experimental models of metastasis include the inoculation of tumor cells into mice so as to assess the property of distant colonization. Intra-cardiac, intra-peritoneal, intra-splenic, the tail vein, and so on, are known routes of tumor cell injection, and have been documented to govern the tissue specificity of metastatic seeding [72]. Recent advances in bioluminescent imaging also permits the non-invasive detection of metastatic seeding by tagged tumor cells, thus ensuring real-time assessment [17,73]. These mouse models include cell-line generated allograft or xenograft systems that often lack the stromal/immune cell heterogeneity associated with the disease; the development of carcinogen-induced and genetically engineered mouse models has allowed cancer biologists to overcome this drawback. 7,12-Dimethylbenz[a]anthracene and azoxymethane-induced skin squamous cell and colorectal carcinoma mouse models, respectively, have been employed in deciphering the mechanisms of carcinogen mediated disease progression [74,75]. Cell lineage specific disease models, generated by Cre-lox approaches, have identified the molecular and cellular cascades contributing to metastasis and discerned disease associated patterns across stochastic cellular events [76,77]. For the improved recapitulation of breast cancer in mice, the mammary fat pad model system was developed so as to ensure the repopulation and manipulation of the mouse mammary gland with human-derived epithelia [78]. Similarly, patient-derived xenografts (PDXs) have been established, which can recapitulate metastatic and organ homing properties similar to the clinical specimen [79]. Fluorescent tagging of lineage-specific tumor cells and the establishment of confetti mouse models permits the tracing, isolation, and characterization of cell populations, thereby systematically dissecting the events involved in development and disease progression [80,81,82,83]. Reporter tags also facilitate CTC isolation and the detection of low frequency tumor cells at secondary sites during the colonization phase of metastasis [79]. Similarly, inducible reporter systems permit the fine tuning of specific molecular events, besides contributing to the spatiotemporal resolution of gene regulatory networks driving metastasis [75].

Functional assays and model systems thus simplify the comprehension of several mechanisms contributing to metastasis. However, the stromal and immune cell populations that facilitate metastasis are often under-represented in classical assays. These cellular interactions include platelet-coated CTCs, which exhibit enhanced survival and immune evasion; cancer associated fibroblasts (CAFs) or tumor associated macrophages (TAMs) in the primary and metastasizing tumor, which facilitate the generation of a supportive niche; and so on. [2,84,85]. Furthermore, the information provided by each assay exhibits cell-type and experimental-system associated context dependency [86]. Hence, an acknowledgement of the inherent shortcomings for each methodology is crucial, prior to the derivation of relevant conclusions, and may necessitate the development of improved protocols.

### 2.3. Scrutinizing Outcomes of Metastasis Assays

Most conventional in vitro assays associate metastasis with migratory and invasive capacities, while the processes of cell dissociation and colonization remain under-represented. Isolated read-outs can misrepresent the metastatic cascade and hinder effective translation of in vitro observations. Herein, we summarize the relevant biological limitations associated with in vitro read-outs (Table S2), and emphasize that a routine re-evaluation of assays is necessary for deriving appropriate clinical inferences.

**Figure 2:**
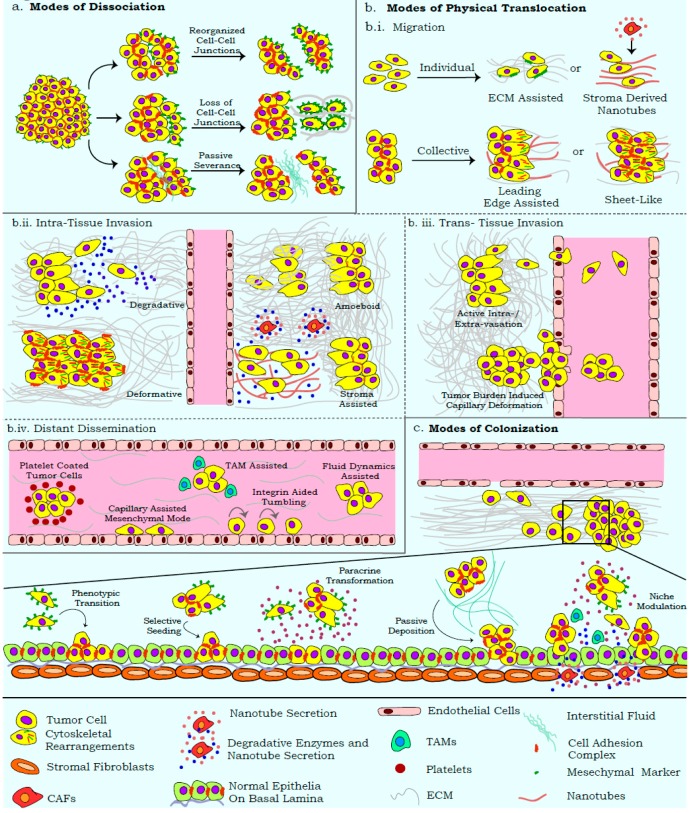
Modalities of metastasis. Metastasis is achieved by a step wise progression of tumor cell dissociation, physical translocation, and colonization. (**a.**) Cell dissociation entails a combination of cell–cell junctional complex rearrangements; phenotypic transitions, like EMT, that result in a loss of epithelial junctions; or the passive severance of cell clusters by virtue of forces exerted by the interstitial fluids. (**b.i.**) Physical translocation involves a myriad of processes, amongst which cell migration is mediated by individual or collective clusters of cells. Individual cells migrate with the aid of ECM or stroma derived nanotubes, whereas collective migration is achieved by an active leading edge and/or by migration of cell sheets. (**b.ii.**) Migrating cells then undergo intra- and trans-tissue invasion by mediating distinct interactions with the ECM and non-transformed cell populations. Intra-tissue invasion involves the degradation and deformation of the ECM, either by the tumor or stromal cells. Alternatively, cells exhibit amoeboid invasion by altering their membrane fluidity so as to squeeze through the ECM with minimal disturbance to the surrounding architecture. (**b.iii.**) Trans-tissue invasion involves the disruption of endothelial linings by virtue of active intra-/extra-vasation or mechanical rupture because of an extensive tumor load. (**b.iv.**) Disseminating tumor cells in circulatory/lymphatic systems can exist either as platelet or tumor associated macrophage (TAMs) coated entities, mesenchymal cells along capillary linings, passively dispersed cell clusters, or exhibit an integrin mediated tumbling similar to cells of the immune system. (**c.**) The final stage of metastases establishment involves the colonization of tumor cells at distant sites mediated by phenotypic transitions, like mesenchymal to epithelial transition (MET), the selective seeding of epithelial cells from heterogeneous clusters, paracrine transformation of secondary site by the tumor cell secretome, passive deposition by interstitial fluids, and modulation of the secondary niche by activation of the tissue stromal compartment.

The effective dissociation of cells from the primary tumor generates varied metastasizing entities (heterogeneous cell aggregates, single mesenchymal cells, and epithelial cell clusters) based on their modes of severance [37,87,88]. Differential molecular programs activated during cell dissociation from the primary tumor endow cells with functional signatures that can influence assay read-outs (Figure 2a). The acquisition of anoikis resistance by these entities is influenced by membrane dynamics, modified secretome, immune/stromal cell recruitment, and forces generated by primary tissue/interstitial fluids; parameters that are often neglected by in vitro assays [36,89,90]. Similar influences also distinguish cell aggregates from spheroids, and demand unambiguous identification approaches for these suspension entities [37]. The re-organization of tissue architecture during metastasis further employs processes that can degrade and/or realign specific ECM components in order to ensure optimal dissemination and colonization (Figure 2b.i) [91,92,93]. While ECM-based assays discern the molecular players of cell adhesion and degradation, there is limited comprehension of the influences from 3D-ECM rearrangement. For example, with minimal biochemical changes, collagens can undergo rearrangement with respect to fiber density and crosslinking, so as to alter cell–ECM interactions [94]. Similarly, degradation assays more commonly quantify the biochemical aspect of cell–ECM interaction, while the mechanical forces that can distort ECM arrangement are largely ignored (Figure 2b.ii) [43,95,96]. Existing assays also disregard the amoeboid mode of invasion, wherein cells exhibit a greater degree of deformability, which facilitates displacement across the ECM with minimal biochemical or mechanical alterations of the matrix [97,98].

In vitro migration and invasion assays present with similar shortcomings, wherein the heterogeneity and mechanics of these processes are excessively simplified and the modalities of translocation are overlooked. Wound closure assays widely imply cell migration, while disregarding the proliferative potential of the wound edge [99]. Similarly, experimental systems often employ scratch and gap closure assays interchangeably, with a complete disregard for the differential biological annotations represented by each method. While “gaps” (cell-free zones) are passively generated by artificial barriers positioned around proliferating cells, scratch assays involve the active disruption of cell monolayers. These differences can influence the activation of differential molecular networks and affect assay outputs [40,41]. Furthermore, trans-well assays restrict the movement of collectively invading cells, because of the pre-defined insert pore size that usually permits the passage of single cells. Similarly, co-culture systems often do not account for the paracrine effects of invading tumor cells on the epithelial/endothelial monolayers; these can activate the trans-differentiation and chemotaxis programs involved in the recruitment of CAFs, TAMs, and so on [84,85]. Most read-outs for migration and invasion also assume an onset of EMT, while the contributions of extra-tumoral cells recruited for their cooperative effects are often disregarded (Figure 2b.ii) [100]. Similarly, the plasticity of migratory/invasive modalities in response to differential ECM density, composition, and arrangement also requires critical scrutiny [101].

While several assays depict the properties associated with cell dissemination, the direct detection of metastatic seeding is not achieved in vitro. Metastasis-associated studies also disregard the stages of intra-/extra-vasation and the associated cellular plasticity that may govern efficacy of the metastatic cascade [102,103]. Importantly, routine in vivo models are usually limited by their inherent immunodeficiency that impacts the heterogeneity and efficiency of metastatic dissemination [104]. Similarly, the ectopic transplantation of tumor cells in mice and the infiltration of mouse stroma in these models make them less suitable for studying the role of tumor microenvironment on metastatic dissemination [104]. Thus, despite the existence of numerous elegant model systems, the complexity of metastasis necessitates regular improvements in assay resolution, and emphasizes the inclusion of recent ideologies when inferring from these outputs.

## 3. Uncoupling the Migration–Invasion–Metastasis Ideology

Tumors hijack several molecular programs associated with organogenesis; hence, concepts derived from developmental systems could elucidate the functional attributes observed during tumor progression and metastasis. Apart from tumor cells, the recruitment of tissue stroma/immune components vastly influences each stage of the metastatic cascade in a context dependent manner. The exclusion of several physiological parameters from experimental systems may stem from the limited familiarity and visualization of such processes, which can limit their collation with clinical observations. In this section, we aim to highlight the alternate physiological mechanisms adopted by tumors that uncouple the traditional migration–invasion–metastasis ideology, and warrant inclusion in routine assays (Figure 2).

The dissociation of cells from the primary tumor is widely attributed to active EMT as well as its molecular manifestations that affect cell adhesion and cytoskeletal complexes [105]; other studies highlight the realignment of adhesion complexes towards the generation of passively disseminating epithelial cell clusters [19]. Passive dissemination presents a challenge for experimental detection, although its contribution to metastasis is undeniable (Figure 2a) [106,107]. The degradative secretome that often accompanies EMT can further lead to the severance of single cells from the primary mass [96]. Tumors are also subject to hydrostatic pressures from organ-specific interstitial fluids that slough off weakly connected proliferative cell masses. Separately, the tumor edge can undergo cytoskeleton mediated delamination as epithelial sheets, a feature also noted during development [108].

Several existing studies associate metastatic efficacy with EMT facilitated migration and invasion [109,110,111]. However, recent reports highlight the differential migratory and invasive capabilities of tumor cells, significantly influenced by the microenvironment (Figure 2b.i). Passively dissociated cells can disperse and seed as proximal metastatic lesions in the absence of active migration/invasion programs [106,112]. Furthermore, efficient cell migration along chemo-/duro-tactic gradients relies on the sustained cellular contacts that underscore the existence of epithelial properties and fortify collective migration as a crucial process in disseminating tumor cells [112]. Recent studies also highlight the role of tumor/stroma cell derived membrane invaginations, termed “nanotubes” that can alter ECM arrangement and serve as directed migratory tracks for cancer cells [113].

The uncoupling of EMT from the metastatic cascade is also evident from reports highlighting the invasion of epithelial cell clusters by virtue of tensile forces exerted on the ECM (Figure 2b.ii) [114,115]. Invasion is also reported to be mediated by an amoeboid transition, which allows cells to squeeze through the ECM, without its biochemical or mechanical alteration [98]. Invasion modalities can be influenced by organ-specific architecture, which presents as an anatomical barrier for metastasis. For example, fenestrated bone marrow sinusoids offer a lower mechanical challenge for invasion, as opposed to the blood–brain barrier [116]. Distal metastasis is further ascertained by the density of lymphatic/blood vessels, and the ability of tumor cells to intra- and extra-vasate; invasive cell clusters capable of disrupting the basal lamina and entering circulation may stay lodged in the blood vessels and fail to extravasate (Figure 2b.iii) [117]. Tumor cells in capillaries or lymphatic vessels ensure survival in response to immune surveillance and hydrostatic pressures by existing as cell clusters or recruiting cells from the hematopoietic lineage [2]. Eventually, cells achieve distant dissemination under the influence of systemic circulatory flow, and can also exhibit migration along the capillary linings by virtue of mesenchymal motility or integrin mediated tumbling (Figure 2b.iv) [118,119].

Several of the above phenomena are reversed during the colonization stage of metastasis, wherein tumor cells re-establish cellular contacts and lodge into a supportive niche (Figure 2c). The reversal of certain mesenchymal properties is thus deemed crucial for colonization, as reports highlight the inability of rigid mesenchymal cells to metastasize, despite in vitro migratory and invasive capabilities [34,120]. Phenotypic plasticity plays a key role in enhancing the adaptability of cells to the altered niche, and may generate a heterogeneous disease at distinct sites in response to varying microenvironments [121,122]. The passive deposition and selective seeding of epithelial tumor cells from heterogeneous metastasizing clusters can dictate the efficacy of metastasis [106,107,112]. Sculpting of the secondary site is also undertaken by tumor cells, wherein paracrine signaling can result in a transformation of the secondary tissue or activate stromal cells, and generate a microenvironment conducive to metastases seeding [100]. Insights into the role of these extra-tumoral cellular components have been reviewed elsewhere [84,85]. Thus, a definitive comprehension of the metastatic cascade necessitates an approach wherein the aforementioned processes can be integrated into informative protocols.

## 4. Visualization of Metastatic Modalities with Real-Time Approaches

**Figure 3:**
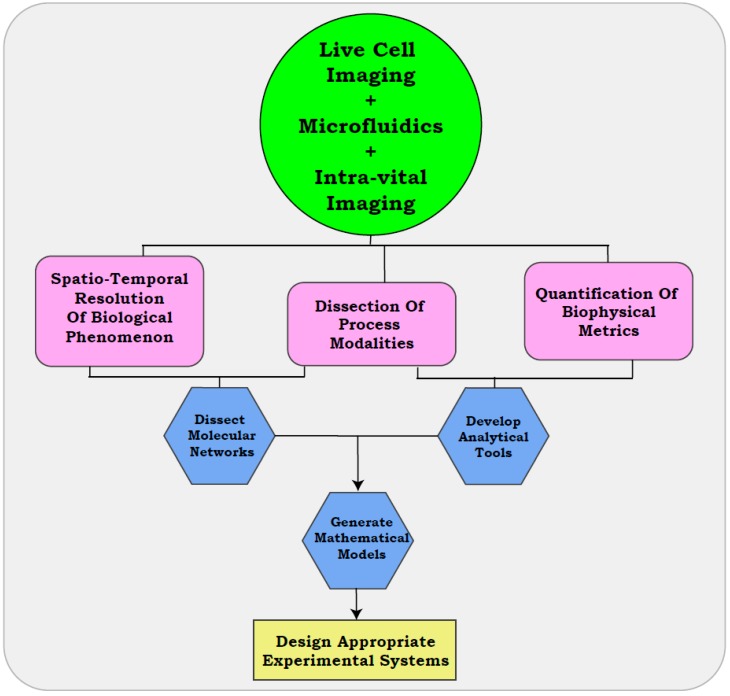
Era of real-time analysis. Routine implementation of real-time approaches in cancer biology can spatio-temporally resolve cellular processes and biological modalities by permitting the extraction of quantitative metrics associated with these states. Outputs from real-time studies can be collated to identify the regulatory networks governing the biological processes, and can be further applied to develop analytical tools/mathematical models for implementation in therapeutic/clinical screens. Such analyses support the design of relevant animal models, recapitulating the in situ patho-physiological parameters of the disease. Corroboration with microfluidic devices further enhances the outputs of these pipelines.

As discussed in previous sections, comprehending the biological heterogeneity of metastasis is often limited by the poor resolution of snapshot assays. Existing assays can provide an enhanced resolution of the cellular functionalities, when coupled with microfluidic approaches, real-time visualization, and intra-vital imaging. These methods can further reform traditional ideologies by including physiologically relevant variables and the development of quantitative parameters to delineate the metastatic cascade (Figure 3).

### 4.1. Use of Microfluidics

Microfluidic devices are recent advances in automation that have been applied in order to elucidate the distinct ECM components, widths of migratory paths, cell adhesion forces associated with collective migration, and the effect of extrinsic fluid pressures, amongst other metrics associated with metastasis [103,123,124,125]. In a recent study, an artificial circulatory system was generated on PDMS (polydimethylsiloxane) micro-capillaries lined with functional endothelial cells. This system was employed to model extra-/intra-vasation modalities under influences of extrinsic fluid pressure [102]. Label-free isolation and characterization of viable CTC clusters from blood samples has been achieved with Cluster-Chip, a microchip technology employing bifurcation traps. CTCs released from the microchip could be subjected to functional and molecular characterization in vitro, to comprehend their role in the metastatic cascade [126]. Similarly, an organ chip bioengineered to mimic the 3D microvascular networks has been effective in capturing and quantifying trans-endothelial migration, seeding, and micro-metastases formation [127]. Similar approaches have also used 3D bio-matrices to generate tissue models for studies pertaining to the interaction and infiltration of cancer cells [128,129,130]. Recently, the co-culture of breast cancer cells and CAFs on a microfluidic device successfully established a 3D-organotypic model to mimic stroma-driven tumor cell invasion. Live cell imaging coupled with transcriptome analysis of the co-cultured cells further discerned the novel molecular targets associated with invasion [131]. The recapitulation of spatio-temporal metabolic adaptations encountered by tumor cells was also achieved with a microfluidic organotypic breast cancer model, and employed to devise therapeutic strategies for targeting hypoxic cells [132].

### 4.2. In Vitro Resolution of Metastatic Modalities with Live Cell Imaging

Independent of microfluidic devices, live cell imaging has enabled the quantification of various functional properties by visually monitoring routine in vitro protocols. The spontaneous detachment of cells from 3D culture models of ovarian cancer was recently visualized with live cell imaging. These observations allowed for the development of in vivo lineage tracing methods, and discerned a collective dissociation of cells from the primary tumor, indicating the onset of metastasis [37]. A recent study from our group, coupling live cell imaging with the in vitro scratch assay, not only discerned the distinct migratory modalities in ovarian carcinoma (CCM versus EMT), but also resolved subtle variations within CCM; this defined CCM as being mediated either through proliferation (passive CCM) or sheet-like migration (active CCM). We quantified the altered migratory modalities in response to extrinsic and intrinsic stimuli, further establishing an improved prototype of the scratch assay [99]. In a separate study, CCM was assessed in the context of durotaxis, wherein live cell imaging successfully quantified the extent and pattern of migration in response to ECM variations [133]. Similar approaches were applied to invasion assays, so as to determine the morphological variations of patient derived spheroids invading into a carboxy–methyl cellulose matrix [134]. Separately, live cell imaging of the migrating cells in a confined micro-pillar array permitted the derivation of distinct quantitative parameters for distinguishing the individual and collective modes of migration [135].

Real-time studies can thus offer an improved comprehension of the bio-mechanical features associated with metastasis that are often overlooked by snapshot assays (Table S3). The corroboration of time-lapse microscopy with trans-well invasion assays could distinguish the degradative, deformative, and amoeboid modes of cell invasion [98,136]. Similar approaches with co-culture systems can delineate the proliferation driven disruption of endothelial linings from active events of intra-/extra-vasation, and in suspension cultures, distinguish the mechanical forces associated with spheroids versus cellular aggregates [38,115,137]. The assay resolution can be further enhanced by employing fluorescent dyes or protein tags that differentially label cell membranes, ECM fibers, cytoskeletal components, and so on. Importantly, the labelling methods adopted for real-time imaging studies must be permissive of the assay systems, and contribute minimal hindrance to the biological process under study [138,139]. Such real-time analysis can also be implemented in high-throughput screens, along with traditional assay outputs, so as to address the effects of growth additives and pharmacological compounds on diverse biological modalities.

### 4.3. Intra-Vital Imaging Assisted Visualization of Cellular Properties

As discussed previously, the limited in vitro recapitulation of the heterogeneity associated with metastasis has compelled cancer biologists to use animal models suitable for varied experimental approaches. The in vivo dynamics of migration and invasion have been previously noted in *C. elegans*, fruit fly, and zebrafish [67]. Recent advances in microscopy and fluorophore chemistry have also permitted the intra-vital single-cell resolution in vertebrate animal models. In a mouse model, intra-vital imaging assisted approaches elucidated the role of Rac1-dependent membrane protrusions in the maintenance of 3D spatial positions of mouse dermal fibroblasts during wound healing [140]. In yet another report, the transplantation of tumor cells into the mouse cerebellopontine angle region coupled with intra-vital imaging successfully developed a model to study the disease progression of vestibular schwannoma [141]. Separately, the intra-vital imaging of early breast carcinoma lesions identified a Her2 driven dissemination program that enables the dispersal of dormant cancer cells to distant organs, prior to the development of a primary tumor [142]. Intra-vital imaging also successfully discerned the significance of orthotopic, as opposed to subcutaneous, tumor models in prostate cancer wherein drastic differences in the overall vascularization and distant metastasis were duly noted [143]. Intra-vital imaging has been aided by confetti mouse models, which express a combination of fluorescent proteins in response to the Cre-recombinase, and permit the visualization of the in vivo heterogeneity inherent to tissue systems [144,145]. Confetti mice have been instrumental in comprehending squamous cell carcinoma progression, wherein the recruitment of the adjacent epithelium by monoclonal papillomas and intra-tumoural invasion by newly generated clones were effectively visualized [146]. Cell of origin studies in castration resistance prostate cancer, employing confetti mice, have previously assigned tumor formation to a population of Bmi1^+ve^ luminal cells in the proximal prostate [83]. The in vivo fate and properties of transplanted corneal epithelium were also resolved with intra-vital imaging, which tracked the fluorescence signals from the confetti tagged donor cells [147].

Other advances in imaging technologies include improved tomography scans, fluorescence lifetime imaging microscopy (FLIM), coherent anti-Stokes Raman microscopy (CARS), and so on, and have been reviewed elsewhere in detail [148,149]. The routine application of such high-resolution approaches may be beneficial towards the development of more informative protocols, the quantification of key biomechanical features, and could overcome several limitations harbored by snapshot studies.

## 5. Quantitative Resolution of Biological Modalities

Examples of real-time assays discussed in previous sections not only emphasize their visual impact, but also highlight the derivation of varied quantitative metrics. The integration of live cell imaging into routine approaches can quantify diverse physiological parameters and enhance assay read-outs. We briefly familiarize readers with recent approaches developed for high-resolution real-time imaging, user-friendly analytical tools, and quantitative variables generated from imaging datasets (Table S3).

Continued technological advances and automation in the field of optics have provided high-resolution microscopes that facilitate the real-time visualization of dynamic biological processes, including the migration of metastatic cancer cells in vivo [150]. Similarly, atomic force microscopy (AFM) and protrusion force microscopy (PFM) have been employed to discern the mechanical forces involved in leukocyte–endothelial adhesion and invadosome–assisted extravasation, respectively [151]. CARS coupled with two-photon imaging has also emerged as a promising approach to describe multiple aspects of the tumor niche and disease progression, with minimal photo-damage to the specimen [73]. Interested readers may refer to several recent articles elaborating on the advances in microscope design, with significant implications in real-time cancer research [148,149].

Datasets generated by real-time imaging present a significant challenge for analysis, thus necessitating familiarity with relevant software and tools. While the adaptability of imaging tools across model systems is beneficial, optimal quantification can only be ensured when high-resolution/-contrast images are provided as inputs. The availability of user-friendly interfaces, improvements with recent plugins, and the development of codes for the customization of outputs enhance the applicability of imaging tools. Currently, a variety of programs facilitate time-lapse image analysis, and are available either commercially, viz., Metamorph (Olympus), Imaris (Bitplane), Zen (Zeiss), Elements (Nikon), Volocity (PerkinElmer), and so on, or as open source platforms, viz., Micro-manager, ImageJ, MotilityLab, and so on. Imaris is specifically useful for 4D data analysis, and can be substituted with the ImageJ post-installation of plugins that facilitate drift correction, volume measurement, free rotation, and so on [152]. COMBImage, a recent computational framework, has facilitated the automated analyses of cell morphology and confluence in cell viability assays [153]. The management and analysis of multi-dimensional imaging data have also been aided by support vector machines (SVM), which have been applied to group pixels for image segmentation, to detect cellular/sub-cellular phenotypes, or to categorize developmental stages at the level of entire organisms [154]. Similarly, mathematical approaches for migration track analysis have been developed [155]. A more comprehensive list of the currently available image processing software and tools is available elsewhere [156,157].

Analytical tools can yield extensive information from imaging datasets, and often confound untrained individuals. The extraction of relevant quantitative metrics must consider the biophysical properties of a system and examine the associative trends with the cellular processes under study. Examples highlighting the selection of quantitative metrics are summarized in Table S3, and include the micro-pillar array-based study, wherein six quantitative parameters extracted from individual cells generated a binary solidification model for describing the interconversion between collective versus individual modes of migration [135], fractal analysis that described the developing dermis as a lattice structure resulting from the dynamic stroma [158], derivation of migration modalities in ovarian cancer cell lines assisted by coupling live cell imaging with the in vitro scratch assay [99], and so on. Additional studies highlight the vast array of quantitative measures derived from real-time imaging datasets, and signify their contributions to an improved understanding of the biological processes [11,151,159,160,161,162].

## 6. The Era of Live Cell Imaging

The simple corroboration of time-lapse microscopy with endpoint assays can reveal the modalities of several biological processes, indicate differential routes of metastasis taken by tumors, and identify the associated molecular pathways (Figure 3). Collation of these datasets can generate multi-parametric simulations that define the biological thresholds associated with diverse cellular functions, and their modulation in response to various cues [163,164,165]. Such models or simulations could aid in the design of appropriate experimental systems that encompass heterogeneous processes contributing to metastasis. Similarly, analytical tools developed with real-time approaches can be integrated into drug development pipelines [166]. In accordance with our statements, a recent study employing intra-vital imaging discerned the crucial role of CD44 driven cell aggregation of CTCs in metastatic dissemination. The study established CD44–PAK2 interaction as a driving event of the process, thus providing clinically relevant information with the aid of visual outputs [167]. While snap-shot assays will continue to provide high-throughput readouts for cell systems, it is crucial that experimentalists acknowledge their limitations when deriving clinically relevant inferences. With the right amount of caution, real-time approaches could herald an “era of live cell imaging” in cancer biology.

Real-time approaches, in conjunction with lineage tracing, may initiate the next phase of resolving cancer heterogeneity, which continues to elude comprehension and is responsible for therapy resistance. In view of recent reports, real-time approaches may decipher functional nuances and provide insight into the concept of “metastability” predicted during phenotypic switches. Real-time dynamics of molecular turn-over can further identify the spatiotemporal alterations of markers over the course of disease progression, and improve clinical interventions. Moreover, intra-vital imaging approaches present an exciting opportunity to tackle a residual minimal disease, which often results in an aggressive relapse and overall poor prognosis. Similarly, extra-tumoral cell populations crucial for disease progression in vivo can be identified and probed in vitro with micro-fluidic and real-time studies. More importantly, the stages of primary dissociation and colonization during metastasis represent a “grey area” of information, which could be effectively resolved with the previously discussed approaches. The delineation of these diverse biophysical features essentially nurtures the model of personalized medicine. A careful combination of appropriate existing assays with live cell imaging can reveal several nuances of the metastatic cascade and reiterate the “seeing is believing” ideology.

## 7. Conclusions

Recent studies reveal a functional uncoupling of several physiological processes previously deemed crucial for successful metastasis. Our own observations associated with the evaluation of in vitro metastasis reflect on the shortcomings of routine assays. For the detection of distinct modalities associated across the metastatic cascade, we suggest the adoption of time lapse imaging in routine assays. This approach has the capability to (i) make a visual impact on biological processes, (ii) identify different routes undertaken by tumor cells towards achieving these functions, and (iii) provide metrics for quantifying these processes. The information obtained from these approaches can be applied towards establishing mathematical models and intuitive inferences that tackle the heterogeneity associated with disease progression. Inherent cellular plasticity that contributes to tissue homeostasis can also be deciphered in the context of pathological conditions, by developing novel assays that combine the ease of traditional approaches with the enhanced resolution of real-time imaging. We also emphasize on the routine re-evaluation of functional read-outs, so as to incorporate recent conceptual developments that improve the comprehension of biological systems. Together, these features are likely to be useful in elucidating the poorly understood areas of disease progression in cancer, and deriving clinically relevant conclusions over the current assays.

## Supplementary Materials

The following are available online at https://www.mdpi.com/2077-0383/8/7/941/s1: Table S1. Metastasis associated functional read-outs. Details pertaining to the setup, quantification, and biological read-out associated with the in vitro and in vivo methods employed to study the metastatic cascade are enlisted. Table S2. Applications and limitations of metastasis associated functional read-outs. Details pertaining to the cellular functionality gauged, stage of metastasis represented, and inherent limitations of the in vitro and in vivo methods employed to study the metastatic cascade are enlisted. Table S3. The real-time approach. Details pertaining to the model systems, relevant instrumentation, quantitative tools and variables, and their biological relevance for recent real-time studies are enlisted. The table includes a summary of a limited number of studies, interested readers may refer to the available literature cited in the text for more information.
