# Supplementary material for: Uncoupling Traditional Functionalities of Metastasis: The Parting of Ways with Real-Time Assays
Source: J Clin Med. 2019 Jun 28;8(7):941. doi: 10.3390/jcm8070941 (PMC6678138; doi:10.3390/jcm8070941)
Supplement: Supplementary file 1 [file jcm-08-00941-s001.zip › Supplementary Table S1.docx]

**Table S1.** Metastasis associated functional read-outs. Details pertaining to setup, quantification and biological read-out associated with *in vitro* and *in vivo* methods employed to study the metastatic cascade are enlisted.

| **Functional Assay** | **Experimental Setup** | **Quantification** | **Readouts** |
| --- | --- | --- | --- |
| ***In Vitro* Assays** | | | |
| Anoikis Resistance | Ultra-low attachment plates | Biochemical / fluorescence based distinction of viable vs. non-viable cells. Commercial kits available for higher resolution | - Percent viability - High percent viability = Higher Anoikis Resistance |
| Spheroid Generation |  | Microscopic evaluation and software assisted image analysis. Resolution may be enhanced by staining or fluorescence. | - Frequency of spheroid generation |
| Trans-Epithelial Resistance | Trans-well inserts, 0.4–0.8µ pore sizes | Probe assisted impedance measurement | - Resistance across a monolayer is quantified - Higher resistance = high integrity of monolayer |
| Dextran Flux | Trans-well inserts, 0.4 – 0.8µ pore sizes. Labelled dextran introduced in culture medium above monolayer | Fluorometric quantification of leaked dextran | - Dextran leakage quantified and correlated with monolayer permeability |
| Co-Culture Invasion | ECM coated culture plates with non-transformed cell monolayer | Microscopic evaluation and software assisted image analysis. Resolution may be enhanced by staining or fluorescence.  *ECM degradation assessed by fluorescence microscopy  *Differential labelling of cells in co-culture/spheroid confrontation assays improves resolution | - Depth of invasion and monolayer disruption is quantified |
| 2D Wound Closure | Monolayer of cells disrupted or grown in presence of barriers to generate cell free zones |  | - Percent wound closure - Enhanced or reduced wound closure in response to extrinsic stimuli is quantified |
| Trans-well Migration | Trans-well inserts, 4 - 8µ pore sizes |  | - Percent migration - Chemotactic effects quantified |
| Trans-well Invasion | Trans-well inserts overlaid with matrigel / ECM components, 4 - 8µ pore sizes |  | - Percent invasion - Invasion through ECM quantified |
| ECM Degradation | ECM/gelatin/collagen coated slides or culture plates |  | - Percent degradation quantified as loss of fluorescence and correlated with MMP activity |
| 2D Cell-Matrix Adhesion | ECM coated plates or arrays in presence / absence of ECM specific antibodies |  | - Percent adhesion - Reduced adhesion in presence of antibody identifies crucial role of each component |
| Spheroid Migration | Ultra-low attachment plates and ECM coated plates |  | - Area of spreading around adhered spheroid |
| Spheroid Invasion |  |  | - Depth of invasion quantified for embedded spheroids |
| Spheroid Adhesion |  |  | - Frequency of adhesion |
| Spheroid Confrontation | Ultra-low attachment plates |  | - Extent of label overlap amongst confronting suspension entities is quantified |
| Soft Agar Colony Formation | Agarose embedded cells gauged for low mechanical tension adhesion associated colonization |  | - Quantification of colony frequency at specific endpoints |
| Organoid Cultures | Ultra-low attachment plates, media and growth factor cocktail |  | - Can be used in above mentioned assays to derive relevant *in situ* conclusions |

| ***In vivo* Assays** | | | |
| --- | --- | --- | --- |
| Circulating Tumor Cells (CTCs) | Microfluidic or flow cytometry based isolation and detection | Frequency of CTCs determined by flow cytometry | - Can be subjected to molecular and functional assays to decipher physical translocation of cells |
| Invertebrate Models of Migration / Invasion | *Dictyostelium discoideum* | Microscopic evaluation and software assisted analysis. Resolution may be enhanced by staining or fluorescence.  *Transgenic lines available for cell lineage tracing in several models  *Teflon Coated Egg Shell Openings Permit Real-Time Monitoring in CAM Assay | - High resolution study of chemotaxis assisted movement |
|  | *Caenorhabditis elegans* |  | - Anchor cell invasion used as a high resolution model for basement membrane invasion |
|  | *Drosophila melanogaster* |  | - Ovarian border cells are an excellent model for collective cell migration - Transgenic lines utilized for studies associated with distant metastatic dissemination |
| Vertebrate (Non-Mammalian) Models for Metastasis | *Danio rerio* |  | - Primordial germ cells serve as a model for amoeboid cell migration |
|  | Ex ovo chick embryo model for metastasis |  | - High resolution model for quantifying tumor cell invasion and metastatic dissemination |
|  | Chick Embryo CAM Assay |  |  |
| Mouse (Mammalian) Models for Metastasis | Tail Vein / Intra-cardiac / Intra-peritoneal / Intra-spelnic Injection Assay | Bioluminscence and eventual tissue section staining based evaluation | - Frequency of micro- and macro-metastases quantified - Possible detection of CTCs |
|  | In vivo Fluorescent Reporters Mouse lines generated to include specific reporter gene cassettes | High resolution study of several physiological processes  *Often lack immune systems and host associated stroma | |
|  | Carcinogen or genetic manipulation mediated progression of diseased states with or without use of clinical samples |  |  |
