# Supplementary material for: Uncoupling Traditional Functionalities of Metastasis: The Parting of Ways with Real-Time Assays
Source: J Clin Med. 2019 Jun 28;8(7):941. doi: 10.3390/jcm8070941 (PMC6678138; doi:10.3390/jcm8070941)
Supplement: Supplementary file 1 [file jcm-08-00941-s001.zip › Supplementary Table S2.docx]

**Table S2.** Applications and Limitations of metastasis associated functional read-outs. Details pertaining to the cellular functionality gauged, stage of metstasis represented and inherent limitations of *in vitro* and *in vivo* methods employed to study the metastatic cascade are enlisted.

| **Stage of Metastasis** | **Functional Assay** | **Cellular Properties Assessed** | **Limitations** |
| --- | --- | --- | --- |
| ***In Vitro* Assays** | | | |
| **Dissociation /Physical Translocation** | Anoikis Resistance | Cell Viability in Suspension | - Neglects survival of cell clusters, immune cell coated CTCs and effects of extrinsic pressures |
|  | Spheroid Generation | Cell Viability in Suspension, Stemness, Cellular Plasticity | - Distinction amongst aggregates and spheroids is neglected |
| **Physical Translocation** | Trans-Epithelial Resistance | Inter-Tissue Invasion. Disruption of Cell-Cell Junctions  (Non-transformed) by Tumor Cells | - Low resolution of cell-cell interaction during invasion |
|  | Dextran Flux |  |  |
|  | Co-Culture Invasion |  | - Neglects effects of cancer cells on co-culture monolayer - Excludes immune component of invasion |
|  | 2D Wound Closure | Cellular Displacement and Proliferation | - Neglects migratory modalities - Erroneous representation of wound closure facilitated by proliferation |
|  | Trans-well Migration |  | - Pore sizes permit single cell passage, discounting the migration / invasion of cell clusters - Neglects invasion across cell layers |
|  | Trans-well Invasion | Invasion Across The Intra-Tissue ECM |  |
|  | ECM Degradation | MMP Enzymatic Activity | - Mechanical ECM remodelling is ignored |
| **Physical Translocation/ Colonization** | 2D Cell-Matrix Adhesion | ECM – Cell Interaction | - Effects of ECM arrangement and density cannot be quantified |
|  | Spheroid Migration | Cellular Displacement and Proliferation | - Neglects migratory modalities |
|  | Spheroid Invasion | Invasion Across The Intra-Tissue ECM | - Neglects invasion across cell layers and effects of ECM arrangement |
| **Colonization** | Spheroid Adhesion | Spheroid – ECM Interaction | - Stromal interaction and circulatory micro-forces are ignored |
|  | Spheroid Confrontation | Inter-Tissue Invasion | - Only applicable for suspension entities. |
|  | Soft Agar Colony Formation | Stemness, Proliferation | - Agarose is a poor representation of ECM complexity |
| **Dissociation/Physical Translocation/Colonization** | Organoid Cultures | Stemness, Cellular Displacement, Proliferation, Invasion | - ECM component is lacking from these models |
| ***In vivo* Assays** | | | |
| **Physical Translocation** | Circulating Tumor Cells (CTCs) | Cell Motility and Invasion | - Cell dissociation and colonization phases of metastatic cascade cannot be studied |
| **Physical Translocation** | *Dictyostelium discoideum* | Cell Motility In Response to Chemotaxis | - Dissimilar morphological features, immune   system, stromal heterogeneity and ECM organization as compared to mammalian systems |
|  | *Caenorrhabditis elegans* | Cell Invasion |  |
|  | *Drosophila melanogaster* | Cell Motility, Proliferation, Cell Invasion |  |
| **Physical Translocation / Colonization** | Danio rerio | Cell Motility |  |
|  | Chick Embryo CAM Assay | Cell Motility, Invasion, Proliferation, Adhesion | - Poor resolution of metastatic cascade |
|  | Ex ovo chick embryo model for metastasis |  |  |
| **Dissociation/Physical Translocation/Colonization** | Tail Vein |  |  |
|  | Mouse (Mammalian) Models for Metastasis | Multiple Biological Functionalities Dependent on The Model Under Study | - Models are either immune compromised or often not subjected to intra-vital approaches of data collection. |
