# Supplementary material for: Uncoupling Traditional Functionalities of Metastasis: The Parting of Ways with Real-Time Assays
Source: J Clin Med. 2019 Jun 28;8(7):941. doi: 10.3390/jcm8070941 (PMC6678138; doi:10.3390/jcm8070941)
Supplement: Supplementary file 1 [file jcm-08-00941-s001.zip › Supplementary Table S3.docx]

**Table S3.** The Real-Time Approach. Details pertaining to model systems, relevant instrumentation, quantitative tools and variables, and their biological relevance of recent real-time studies are enlisted. The table includes a summary of a limited number of studies, interested readers may refer to available literature cited in text for more information.

| **Cellular Property** | **Model System** | **Instrumentation** | **Softwares/Programs/Tools** | **Variables Quantified** | **Biological Relevance of Variables** |
| --- | --- | --- | --- | --- | --- |
| Viability (Chantzi *et al*, 2018) | Glioblastoma multiforme cell lines | Phase-contrast time lapse imaging system | COMBImage | (1) Time evolving morphologies;  (2) Confluence; | (1), (2) Synergistic effect of anti-cancer agents on cell viability |
| Morphology, proliferation, motility (Huang *et al*, 2018) | Osteosarcoma cell lines | Phase-contrast time lapse imaging system | QPI software | (1) Object size;  (2) Cell numbers;  (3) Migration trajectory | (1) Changes in cell morphology during;  (2) Proliferative index;  (3) Cell Motility |
| Migration (Varankar & Bapat, 2018) | Ovarian cancer cell lines | Confocal laser scanning microscope | FIJI, MATLAB | (1) Displacement;  (2) Velocity;  (3) Frequency of nearest neighbours | (1) – (3) Modality of migration |
| Migration (Wong *et al*, 2014) | Breast cancer cell lines | Inverted fluorescence microscope | Cell Profiler, MATLAB | (1) Mean Square Displacement (MSD);  (2) Velocity;  (3) Frequency of nearest neighbours;  (4) Sinuosity;  (5) Tortuosity | (1) – (5) Modality of migration |
| Migration (Vedula *et al*, 2014) | HaCaT Cells | Inverted fluorescence microscope, Atomic Force microscope | ImageJ, MATLAB | (1) Particle Image Velocimetry (PIV)  (2) Migratory Path  (3) Curvature of Epithelial Bridge | (1), (2) Migratory Pattern and Directionality  (1) – (3) Behaviour of epithelial bridges and mechanical Properties |
| Migration (Lee *et al*, 2016) | Breast cancer cell lines | Confocal microscope | ImageJ, MATLAB | (1) PIV;  (2) Finite-time Lyapunov exponents;  (3) Fluctuation time scales | (1) – (3) Migratory patterns |
| Migration (Sunyer *et al*, 2016) | Breast cancer cell lines | Atomic Force mircoscope | Gwyddion | (1) Young’s modulus;  (2) Gel displacement;  (3) Tension; (4) Traction;  (5) Tactic index; (6) Speed;  (7) Migration track | (1) – (4) Changes in cell adhesion substrate during migration  (5) – (7) Migratory pattern |
| Tumor Cell Detachment (Al Habyan *et al*, 2018) | Ovarian cancer cell line derived spheroids | Confocal microscope | ZEN, Fiji | (1) Positional co-ordinates of differentially labelled spheroids;  (2) Fluorescence intensities of spheroid confrontation cultures | (1), (2) Modality of cellular detachment involved in generation of cellular entities in ovarian cancer ascites |
| Invasion (Zajac *et al*, 2018) | Patient-derived colorectal cancer spheroids | Spinning disk confocal microscope | ImageJ, Cellsens Dimension | (1) Spheroid centroid position;  (2) Centroid displacement;  (3) Speed;  (4) Spheroid polarity;  (5) Sphericity;  (6) Shape factor;  (7) Perimeter | (1) – (3) Spheroid motility;  (4) – (7) Morphology of the spheroid invading edge |
| Migration and protrusion (Sun *et al*, 2017) | *Drosophila* germ band | 2-Photon confocal microscope | FIJI, Packing Analyzer, Cytoscape, | (1) Front-Back polarity;  (2) Length between Dorsal-Ventral (DV) cells;  (3) Speed of DV migration;  (4) Clustering coefficients;  (5) Modified kissing circle algorithm | (1), (2), (3) DV cell migration;  (4) Rosette cluster organization during germ band extension;  (5) Protrusions from DV cells |
| Migration (Sánchez-Higueras & Hombría, 2016) | *Drosophila* ring gland | Spinning disk confocal microscope | ImageJ, Imaris | Positional co-ordinates of GFP, mCherry, Gal4-lacZ tagged cells | Direction and temporal dynamics of ring gland primordia migration |
| Membrane dynamics (Marsh *et al*, 2018) | Fibroblasts in mouse dermis (young *vs.* old) | Coherent 2 photon confocal microscope | FIJI, Imaris, MATLAB tools | (1) Image correlation coefficients;  (2) Membrane coverage;  (3) Voronoi diagrams;  (4) Collagen intensity | (1) Comparison of image similarity to detect cell movement;  (2) Frequency and length of membrane protruions;  (3) Cell density In tissue of interest;  (4) ECM contribution |
| Stem cell renewal and quiescence (Cockburn *et al*, 2018) | Mouse dermis | 2-Photon confocal microscope | Imaris, MATLAB | (1) Positional co-ordinates of mCherry, CFP tagged cells;  (2) Cell area;  (3) Timescales of self-renewal/differentiation | Self-renewal and differentiation of stem cell populations |
| Fibroblast migration (Jiang *et al*, 2018) | TmG transgenic mouse | Confocal laser scanning microscope | Imaris, ImageJ | (1) Cell tracks;  (2) Fractal Dimensions;  (3) Lacunarity | (1) Migratory patterns of fibroblast sub-populations;  (2), (3) Complexity and heterogeneity of the developing dermis |
| Intravasation (Harper *et al*, 2016) | Tumor cell injected mouse mammary gland | 2-Photon confocal microscope | Imaris, ImageJ | Positional co-ordinates of CFP, tagged cells and 155kDA dextran-labelled vasculature | Surface reconstruction of tumor cell intravasation into capillaries |
| Tumor biology (Lee *et al*, 2015) | MMTV-PyMT mouse | CARS and 2-photon microscope | Imaris, ImageJ | (1) Positional co-ordinates of differentially labelled tumor cells, mouse epithelia, RBCs and vasculature;  (2) Collagen intensity;  (3) RBC migration tracks | (1) Progression of the tumor in the mammary gland and contribution of host components to its growth;  (2) Association of ECM with tumor growth;  (3) Development of functional vasculature in the tumor |
